# Supplementary material for: Integrated analysis of the aqueous humor microbiome and lens capsule transcriptome in high myopia cataract: a pilot study
Source: Front Med (Lausanne). 2026 Jun 16;13:1845205. doi: 10.3389/fmed.2026.1845205 (PMC13314463; doi:10.3389/fmed.2026.1845205)
Supplement: Supplementary file 3 [file Data_Sheet_1.zip › 5.Association/randomforest/C372089/RandomForest.pdf]

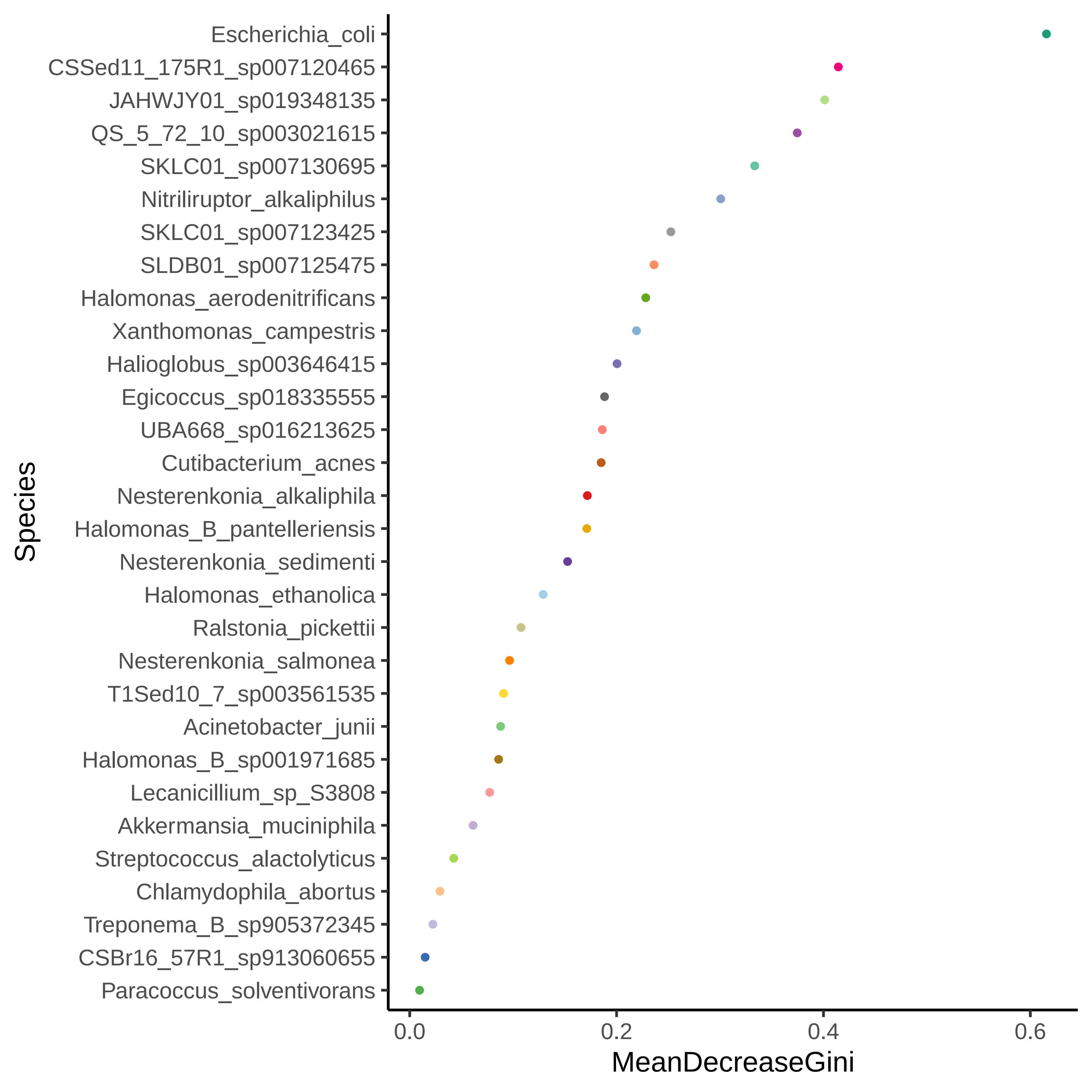

Escherichia_coli
CSSed11_175R1_sp007120465
JAHWJY01_sp019348135
QS_5_72_10_sp003021615
SKLC01_sp007130695
Nitriliruptor_alkaliphilus
SKLC01_sp007123425
SLDB01_sp007125475
Halomonas_aerodenitrificans
Xanthomonas_campestris
Halioglobus_sp003646415
Egicoccus_sp018335555
UBA668_sp016213625
Cutibacterium_acnes
Nesterenkonia_alkaliphila
Halomonas_B_pantelleriensis
Nesterenkonia_sedimenti
Halomonas_ethanolica
Ralstonia_pickettii
Nesterenkonia_salmonea
T1Sed10_7_sp003561535
Acinetobacter_junii
Halomonas_B_sp001971685
Lecanicillium_sp_S3808
Akkermansia_muciniphila
Streptococcus_alactolyticus
Chlamydophila_abortus
Treponema_B_sp905372345
CSBr16_57R1_sp913060655
Paracoccus_solventivorans
Species
0.0
0.2
0.4
0.6
MeanDecreaseGini
